# PP79 Challenges With Integrating Early-Stage Cancer Trial Endpoints Into Economic Models: Review Of Canadian And International HTA Recommendations

**DOI:** 10.1017/S0266462324002484

**Published:** 2025-01-07

**Authors:** Jaclyn Beca, Cherry Chan, Eon Ting

## Abstract

**Introduction:**

Therapies for early-stage cancers demonstrate clinical benefit with early endpoints that measure delayed or avoided disease recurrence, and survival benefits take years to confirm. Economic evaluations for health technology assessment (HTA) require assumptions about long-term benefits to project lifetime disease trajectories. We examined economic modeling approaches used in HTA for adjuvant/neo-adjuvant therapies to understand challenges, explore patterns, and identify opportunities for methodological improvements.

**Methods:**

We included drug indications with Canadian Agency for Drugs and Technologies in Health (CADTH) reimbursement recommendations as of November 2023 for adjuvant/neoadjuvant treatment of early-stage solid tumors. We collected recommendation outcomes and details of submitted clinical and economic evidence. Adapting prior work and focusing on threats to validity arising from equivocal methodological challenges, we classified issues raised in the economic review, particularly those related to surrogacy, treatment pathways, and long-term benefit assumptions (extrapolation, duration of benefit, and cure). We made comparisons with respect to the approaches to long-term benefit assumptions and treatment pathways for drug indications that also had appraisals by the National Institute for Health and Care Excellence (NICE, UK) and the Pharmaceutical Benefits Advisory Committee (PBAC, Australia).

**Results:**

Seventeen drug indications for adjuvant/neoadjuvant treatment of solid tumors were included. Reimbursement was recommended in Canada for 83 percent of drug indications, all were reviewed and recommended in the UK, and 11/15 (73%) have been recommended in Australia. Assessments described overall survival (OS) as immature, but interim OS data appeared to be supportive. There was considerable variability in approaches to long-term benefit assumptions by both submitters and reviewers. Modifications to the assumptions were made in two-thirds of reviews before acceptance by HTA. There was notable inconsistency in approaches to handling treatment-waning, while cure assessment time greater than or equal to five years from initiation was consistently considered appropriate by reviewers.

**Conclusions:**

While all assessments recognized immature OS data, positive reimbursement recommendations were common. There was important variability in application of methods to estimate long-term treatment effectiveness, particularly determining appropriate, evidence-informed assumptions for duration of benefit and cure. This research can support guidance, methodological advancements, and consensus-building to appropriately capture benefits and assess uncertainties for treatments in early-stage cancers with more consistency.

